# Supplementary material for: Shielded soft force sensors
Source: Nat Commun. 2022 Aug 9;13:4649. doi: 10.1038/s41467-022-32391-0 (PMC9363457; doi:10.1038/s41467-022-32391-0)
Supplement: Supplementary file 1 — Supplementary Information [file 41467_2022_32391_MOESM1_ESM.pdf]

Shielded Soft Force Sensors

*Bekir Aksoy[1,2], Yufei Hao[1,2], Giulio Grasso[1], Krishna Manaswi Digumarti[1], Vito Cacucciolo[1], and Herbert Shea[1]**

[1]Soft Transducers Laboratory (LMTS), Ecole Polytechnique Fédérale de Lausanne (EPFL), Neuchâtel 2000, Switzerland.

[2]These authors contributed equally: Bekir Aksoy, Yufei Hao
*e-mail: herbert.shea@epfl.ch

Supplementary information includes the following materials

Supplementary note 1: Deformation of empty vs. liquid-metal filled channels

Supplementary note 2: Candidate sensor designs for shear and normal force measurements

Supplementary note 3: The effect of shielding and the stiffness of the layers on the sensing capacitances

Supplementary note 4: Capacitive coupling between normal and shear deformation

Supplementary Figures 1-15

Supplementary Table 1

Supplementary References

Supplementary note 1: Deformation of empty vs liquid-metal filled channels

Liquid-metals can undergo large deformations while maintaining their electrical conductivity. They require however encapsulation for most of the applications. Due to their incompressibility, the encapsulation stiffens the structure if it is not designed properly. In our design, some of the channels are filled with the liquid-metal eutectic gallium-indium (EGaIn) to serve as the electrodes. The remaining channels are intentionally left empty in order to provide available space for the displacement of the liquid-metal when the sensor is pressed. These empty channels compensate for the incompressibility of the liquid-metal and of the elastomer.

Supplementary Fig. 2 compares the deformation profiles of two designs: 1) one with all channels are empty and 2) one with some channels are filled with liquid-metal while the remaining channels are empty. The results are obtained using COMSOL Multiphysics. In both simulations, the bottom of the structures is fixed and a uniform pressure is applied to the top surface. The structures deform as shown in Supplementary Fig. 2. Since the channels that are filled with liquid-metal are incompressible, these channels have slightly different deformation profiles; the liquid-metal pushes the lower resistance silicone wall (towards empty channels) when the sensor is pressed.

Supplementary note 2: Candidate sensor designs for shear and normal force measurements

We use COMSOL Multiphysics to optimize the layout of electrodes and of air pockets for sensitivity to normal and shear forces. The candidate designs are shown in Supplementary Fig. 3. In all simulations, the silicone layer is made of Sylgard 186 and has a Young's modulus of 750 kPa. The stretchable electrodes and shielding are assumed to be made of the same material as silicone layers. The liquid-metal channels are defined as incompressible, i.e., their volume is conserved.

Three parameters are considered when choosing between the designs; initial capacitance in the undeformed state, the total change in capacitance per unit change in normal force and the total change in capacitance per unit change in shear force (see Supplementary Table 1). Initial capacitance needs to be in a suitable range (< 17 pF) for it to be measured by the hardware used. Designs with higher changes in capacitance when a force is applied have higher sensitivity and are therefore considered to be better than the rest.

The layout with the best performance across the three parameters is one (design #5) in which the top row of electrodes is horizontally shifted with respect to the bottom row by half the channel width. This misalignment of the vertical walls reduces the effective mechanical stiffness of the and therefore amplifies the deformation under an external load (easier to compress the channels) which eventually enhances the sensing performance.

Supplementary note 3: The effect of shielding and the stiffness of the layers on the sensing capacitances

The effect of shielding is analyzed using COMSOL Multiphysics (see Supplementary Fig. 4b). This makes sensing capacitance 11 times more sensitive than parasitic capacitance for a given load. When the passive region is more deformable, parasitic capacitance changes rapidly (see Supplementary Fig. 4b). This is simulated using softer material. In this scenario, the ratio between the sensing and parasitic capacitances decreases to 1.8. Due to the very symmetric design and perfect loading condition in the simulations, we don't see any disturbance in

the sensing electrodes due to this increased parasitic capacitance. In the real devices, however, we observe a decrease in the sensing capacitances when the passive region had comparable stiffness as the sensing region. Therefore, the sensors are designed to have very high capacitance changes for the sensing capacitances and negligible change for the parasitic ones.

Supplementary note 4: Capacitive coupling between normal and shear deformation

Supplementary Fig. 9 shows the cross-section of the simple capacitive force sensor concept based on the parallel plate scheme. The sensor is made of a deformable block of dielectric material of dielectric permittivity *ε.* Three planar electrodes of negligible thickness are placed inside the block at a distance *t*, one symmetrically overlapping the other two over a *w* length. The system develops in the out-of-plane direction by a length *b*. From theory [1], the capacitances between the central (bottom) electrode and each top electrode in the undeformed configuration, namely $C_{L,0}$ and $C_{R,0}$, are given by:

$$C_{L,0} = C_{R,0} = \varepsilon \frac{w\, b}{t} \qquad (1)$$

Let's suppose that a uniform load is applied to the top surface of the sensor as shown in Supplementary Fig. 9, which is resting on a flat surface. We can hypothesize that the sensor deforms homogeneously, causing the top electrodes to slide over the bottom one by an amount Δu and to decrease the gap width by Δt. The capacitance variations caused by the applied biaxial load $\Delta C_{L,0}$ and $\Delta C_{R,0}$ can be written as:

$$\Delta C_L = \varepsilon \frac{(w - \Delta u)b}{t-\Delta t} - \varepsilon \frac{w\, b}{t} = \varepsilon b \left(\frac{t(w-\Delta u)-w(t-\Delta t)}{t\,(t-\Delta t)}\right) = \varepsilon b \left(-\frac{\Delta u}{t-\Delta t} + \frac{\Delta t}{t(t-\Delta t)}\right) \qquad (2)$$

$$\Delta C_R = \varepsilon \frac{(w+\Delta u)b}{t-\Delta t} - \varepsilon \frac{w\, b}{t} = \varepsilon b \left(\frac{\Delta u}{t-\Delta t} + \frac{\Delta t}{t(t-\Delta t)}\right) \qquad (3)$$

Effects of applied normal and shear force can be estimated by evaluating sum and difference of the two capacitances:

$$\Delta C_R + \Delta C_L = 2\varepsilon b \left(\frac{\Delta t}{t(t-\Delta t)}\right) \qquad (4)$$

$$\Delta C_R - \Delta C_L = 2\varepsilon b \left(\frac{\Delta u}{t-\Delta t}\right) \qquad (5)$$

Whereas the sensor compression can be obtained directly from measuring the sum of the capacitance, the response to shear deformation will also depend on the applied compression, thus representing a coupling between normal and shear force effects.

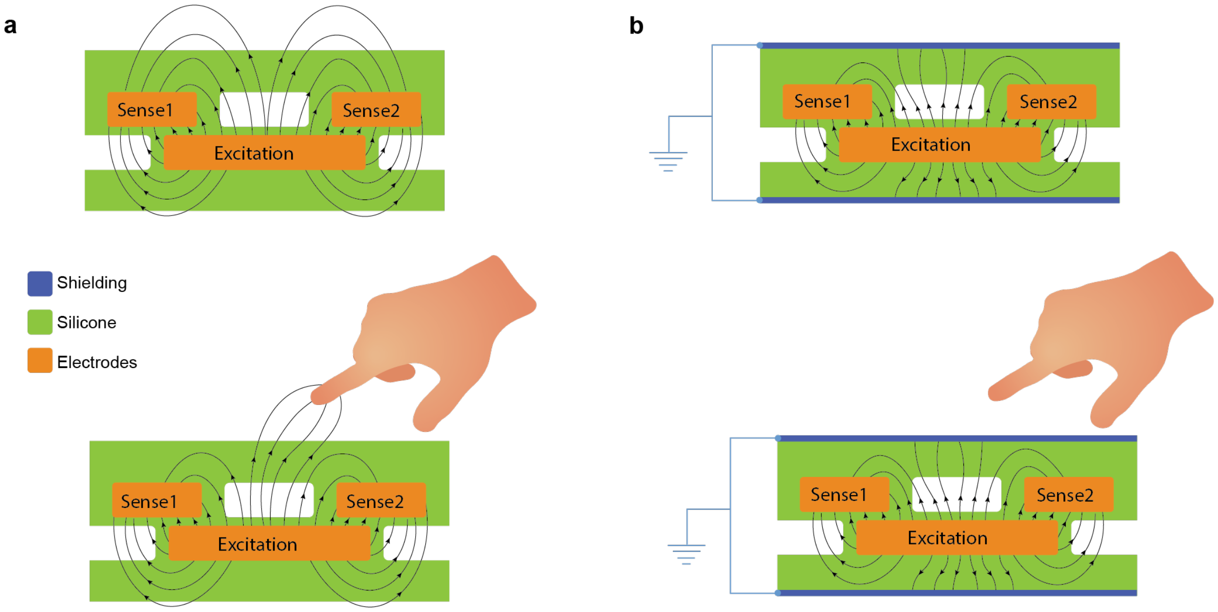

Supplementary Figure 1**. The distribution of electric fields in the unshielded and shielded sensor**. **a** The distribution of the field is interrupted by an approaching finger in the unshielded sensor. **b** The grounded shielding maintains the initial electric field distribution and therefore is not affected by approaching object.

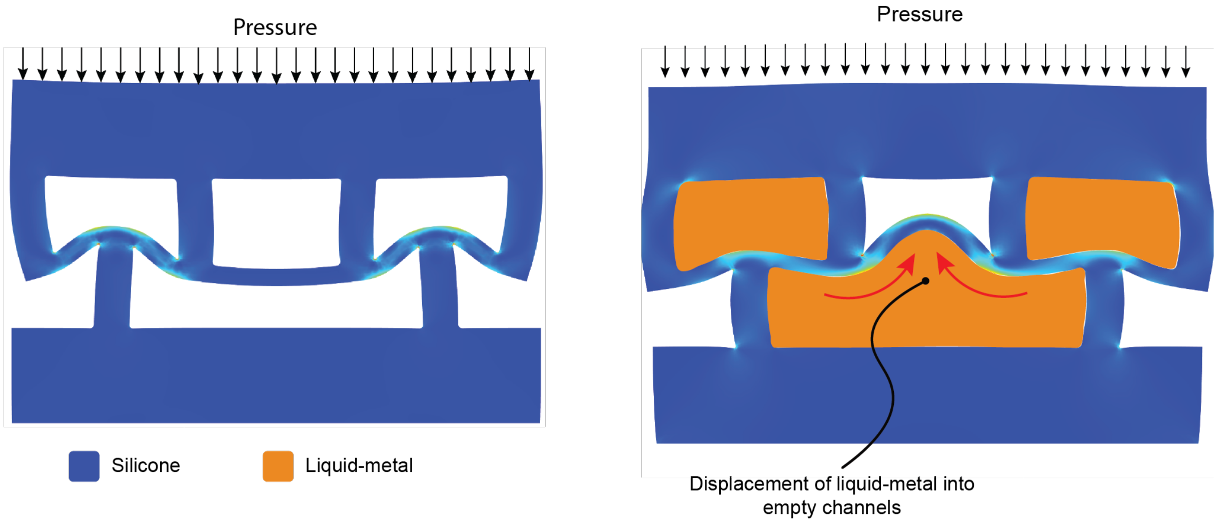

Supplementary Figure 2. **Comparison of structural deformations when the channels are completely empty and when they are partially filled with liquid-metal.** Since the LM is incompressible, the channels have slightly different deformations. In our sensor design, some of the channels are left empty to accommodate available space for the LM displacement when the sensor is pressed.

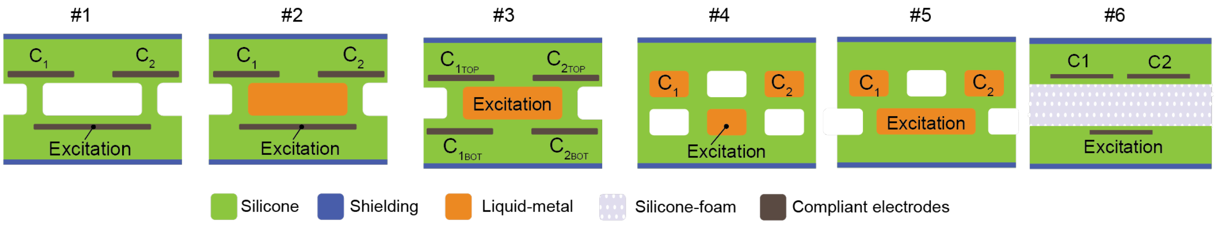

	#1	#2	#3	#4	#5	#6
Total height	1.90	1.90	1.90	2.20	2.20	0.80
Total width	2.65	2.65	2.65	3.05	3.05	5.00
Channel width	1.50	1.50	1.50	0.50	1.40 & 0.5	5.00
Channel height	0.50	0.50	0.50	0.50	0.50	0.20
Vertical wall	0.20	0.20	0.20	0.20	0.20	-
Horizontal wall	-	-	-	0.10	0.10	-
Depth	15.0	15.0	15.0	15.0	15.0	15.0
Overlap	0.625	0.625	0.425	-	0.275	0.50

Dimensions are in mm.

Supplementary Figure 3. **Candidate sensor designs evaluated using COMSOL.** The dimensions of each design are listed below. The designs are compared based on their initial capacitance, their capacitance change per normal force, and capacitance change per shear force.

Supplementary Table 1. **Performance comparison between different sensor designs.** The values are shown for one sensing unit. *For the foam design, it is difficult to get the correct dielectric permittivity to simulate. For this sensor we use the measured values.

Design #	Initial Capacitance (pF)	$\Delta C/F_{normal}$ ($fFmN^{-1}$)	$\Delta C/F_{shear}$ ($fFmN^{-1}$)
1	0.325	0.400	0.180
2	1.364	1.000	0.190
3	4.700	3.000	0.580
4	0.530	1.250	0.050
5	1.970	1.200	0.950
6*	4.30	4.35	0.77

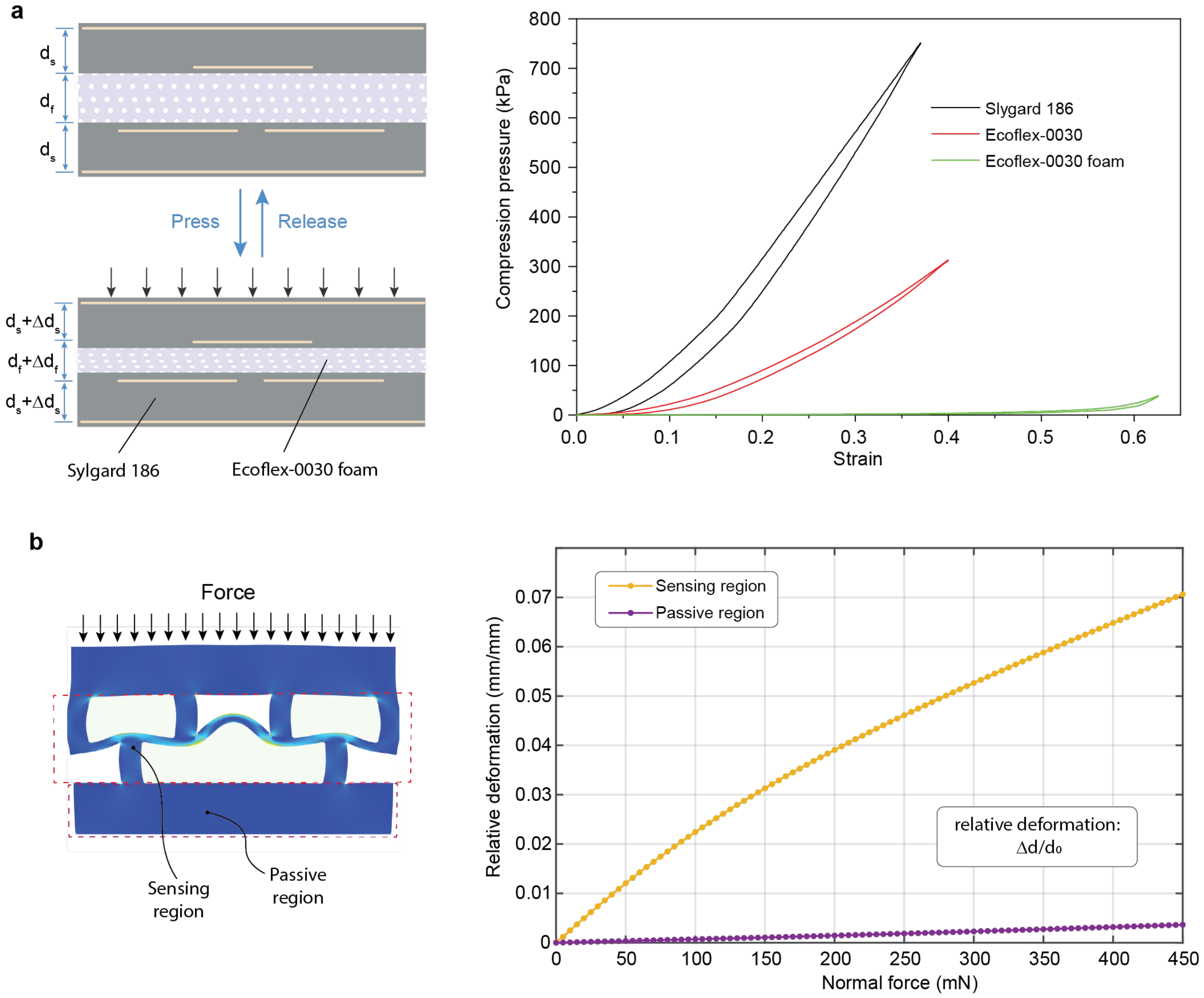

Supplementary Figure 4. **Designing functional layers with different stiffness to obtain high force sensitivity. a** Cross-sections of the silicone-foam sensor showing the sensing and passive regions. The sensing region is the more deformable section of the sensors and undergoes large deformation under an external loading. The measured compressive stress and strain of materials used in the silicone-foam sensors. **b** The relative deformation (as proxy for deformability) of the active and passive regions in the LM sensor is plotted as a function of the applied normal force. For small forces (< 100 mN), the ratio between these regions is 30 and decreases down to 20 at 450 mN of force, showing the stiffening of the sensing region. Thanks to this stiffening, the sensors can survive at very high forces (> 20 N).

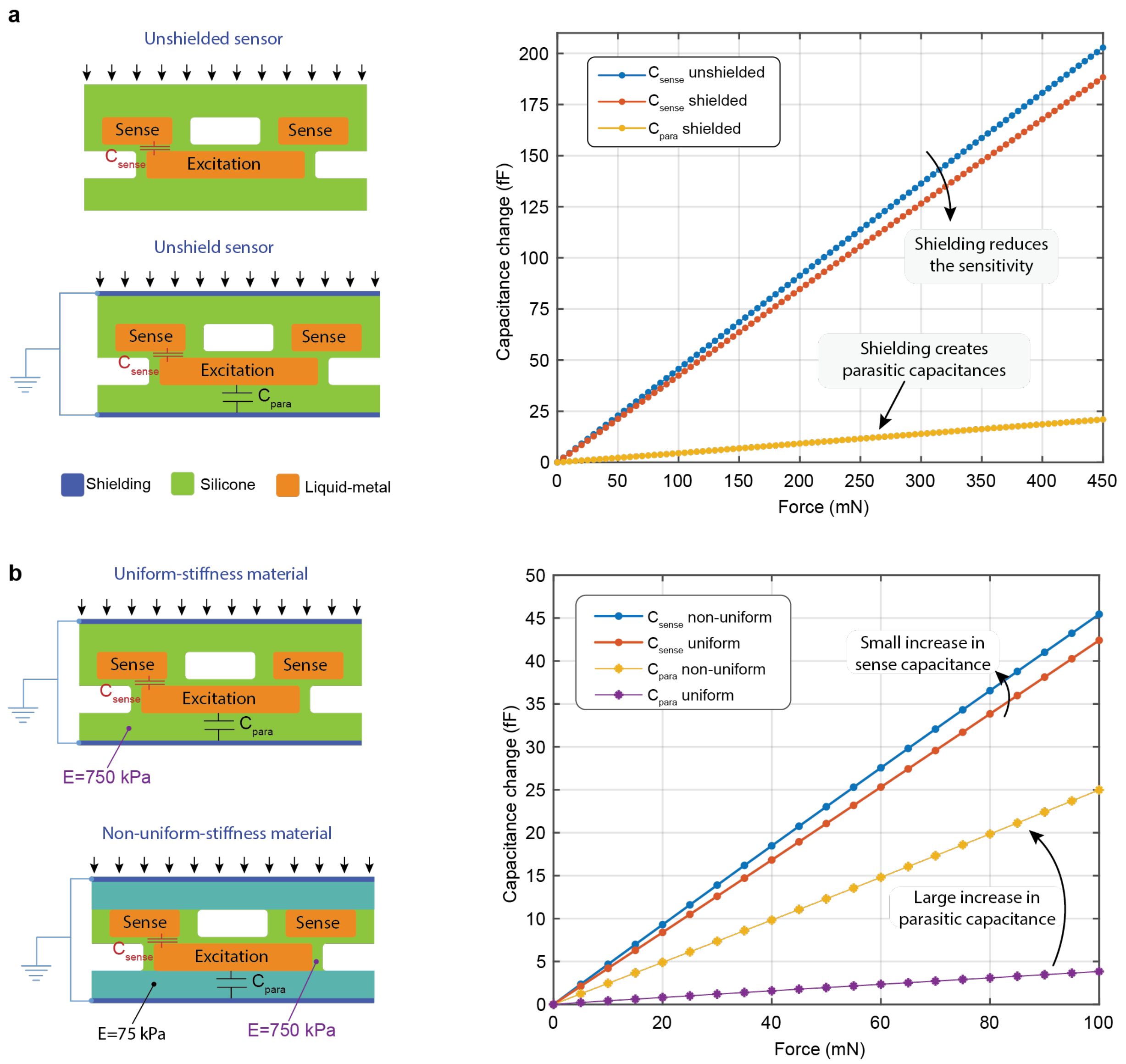

Supplementary Figure 5. **The effect of shielding and the stiffness of the layers on the sensing capacitances. a** The shielding creates additional parasitic capacitances and decreases the sensor sensitivity, e.g. from 0.45 fFmN^{-1} to 0.41 fFmN^{-1}. **b** Having comparable deformability for sensing and passive regions increases the parasitic capacitances.

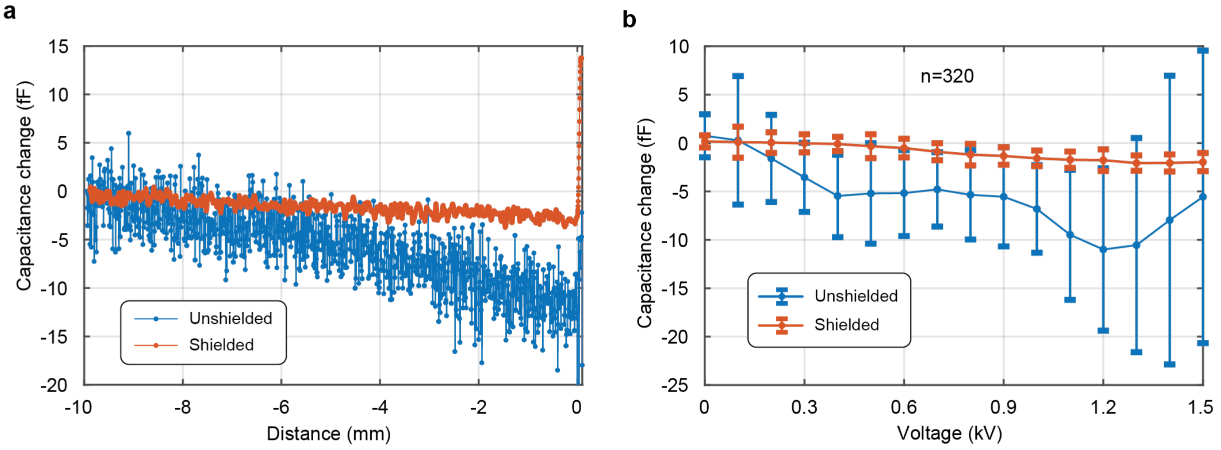

Supplementary Figure 6**. Shielding performance of silicone-foam sensors. a** The capacitance changes of the shielded and unshielded silicone-foam sensors when approaching with a metal plate. **b** The capacitance changes due to the fringing electric field of a nearby electroadhesive patch that is operated at different AC voltages. In both scenarios the shielded sensors are not affected by these interferences whereas the unshielded sensors are very vulnerable to these changes. Error bars represent the mean ± 1 standard deviation over 20 s of measurement (n = 320 for each voltage step).

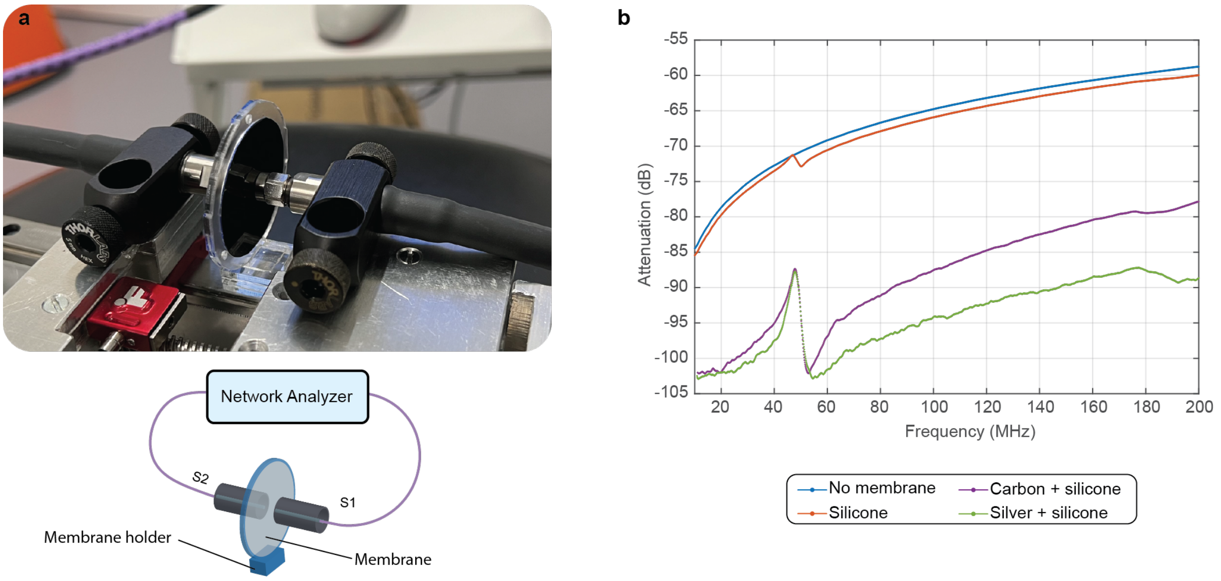

Supplementary Figure 7. **Measurement of transmission of different shielding layers using a network analyzer (E5071C from Agilent Technologies). a** Photograph and schematic describing the experimental setup to measure the shielding properties of circular thin films. Two probes of the network analyzer are clamped with a spacing of approximately 0.5 mm. The film to be tested is placed between them. The S_{21} parameter is measured while the frequency is swept from 10 MHz to 200 MHz. **b** Signal attenuation for different materials plotted vs frequency. The case of: no membrane between the probes, bare silicone membrane, carbon-based silicone composite membrane, and silver-based silicone membrane are compared. The silver-based shielding performs the best, attenuating the signal by 19 dB to 30 dB compared to bare silicone, whereas the carbon-based shielding reduces by 18 dB to 20 dB compared to bare silicone.

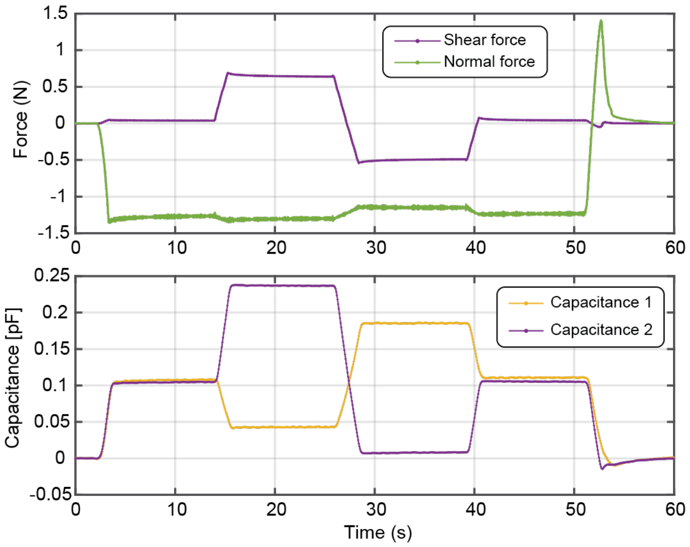

Supplementary Figure 7. **The change of the capacitances when the normal and shear forces are applied to the sensor.** The top graph plots the normal and shear forces applied to the sensor. The sensor is first compressed by applying 1.31 N normal force. It is then additionally deformed in the shear direction with a shear force of 0.65 N. The shear force is then flipped while the normal force is kept constant (+normal - shear). Finally, the shear force and normal force are removed. The time evolution of the capacitances is plotted in the bottom graph as the sensor goes through these steps. When the normal force is applied, both capacitances increase. The shear force however, changes the capacitances in the opposite ways, e.g. decreasing ΔC_1 while increasing ΔC_2. Similarly, flipping the shear force direction increases ΔC_1 and decreases ΔC_2.

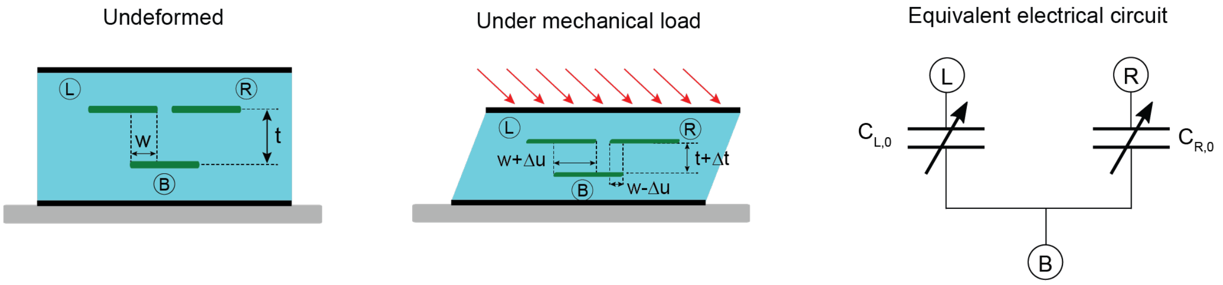

Supplementary Figure 9. **Schematic representation of the electric coupling between normal and shear force effects.**

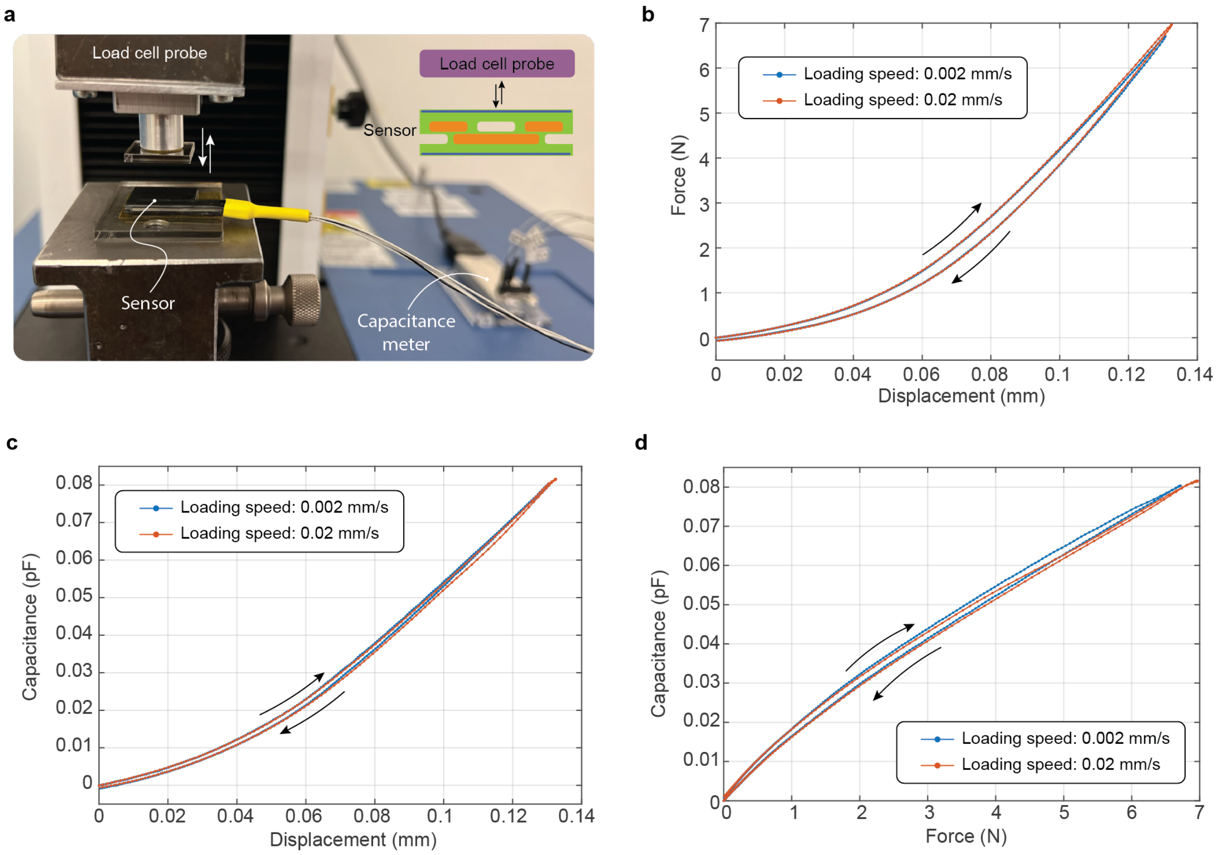

Supplementary Figure 10**. Hysteresis of force and of capacitance during loading and unloading. a** Photograph and schematic of the experimental setup used for hysteresis analysis. **b** The graph plots force vs. displacement of the liquid metal sensor taken at two different speeds: 0.002 mms^{-1} and 0.02 mms^{-1}. The sensor has a small degree of elastic hysteresis of 5% and it is the same for the tested loading speeds. **c** The capacitance change during loading and unloading plotted against the applied displacement, showing 2% of hysteresis. The capacitance hysteresis is the same for both loading rates. **d** The capacitance change plotted as a function of the applied load. The capacitance change shows a small difference between loading and unloading curves and is the same for different loading speeds.

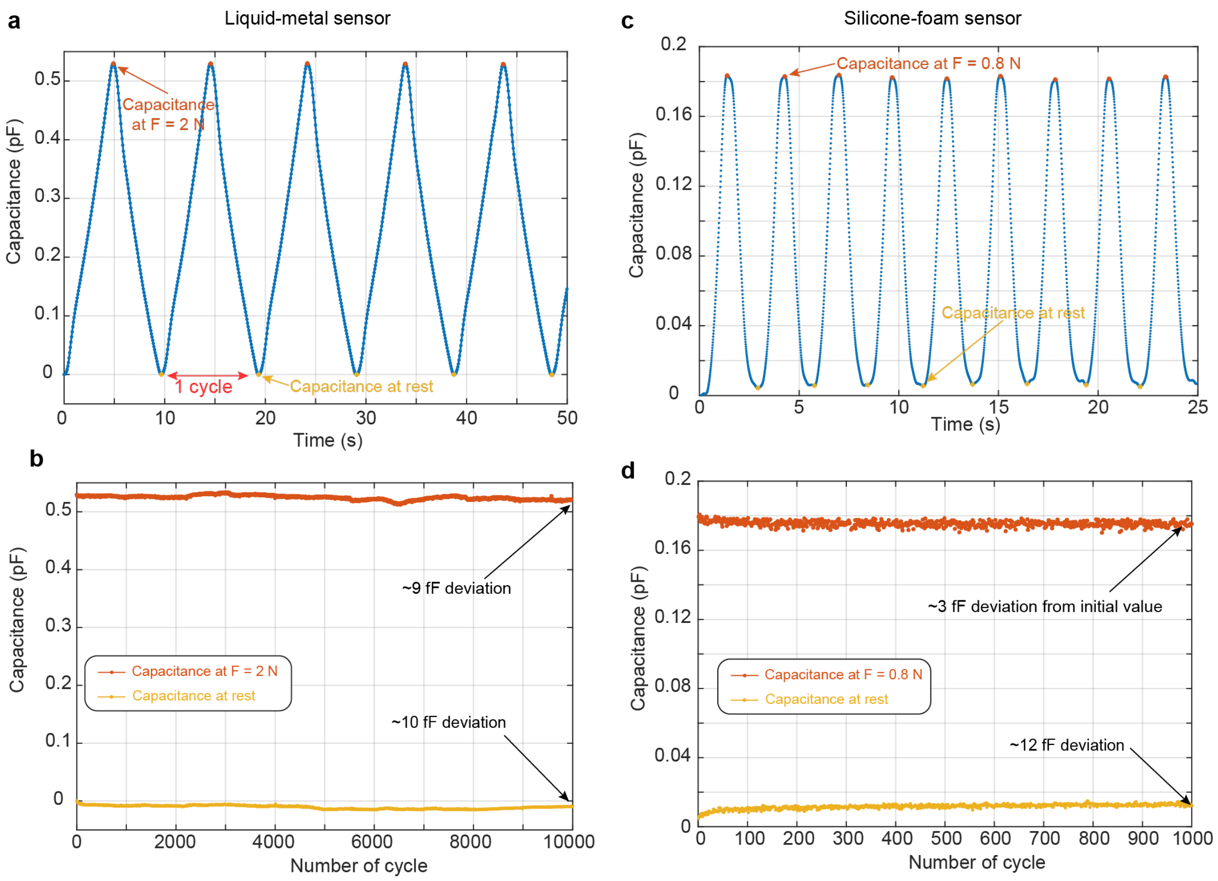

Supplementary Figure 11. **Performance of the sensors under cyclic loading. a** Capacitance change of the liquid metal (LM) sensor under an applied force of 2 N for the first five cycles. **b** Capacitance change of the LM sensor under an applied force of 2 N and at 0 N vs. cycle number for 10^4 cycles. The capacitance difference between the first and last cycles is less than 10 fF, corresponding to <35 mN force. **c** The capacitance of the silicone-foam (SF) sensor under an applied force of 0.8 N vs. time for the first nine cycles. **d** Capacitance change of the SF sensor under an applied force of 0.8 N and at 0 N vs. cycle number for 103 cycles. The capacitance difference between the first and last cycles is less than 12 fF, corresponding to <53 mN force.

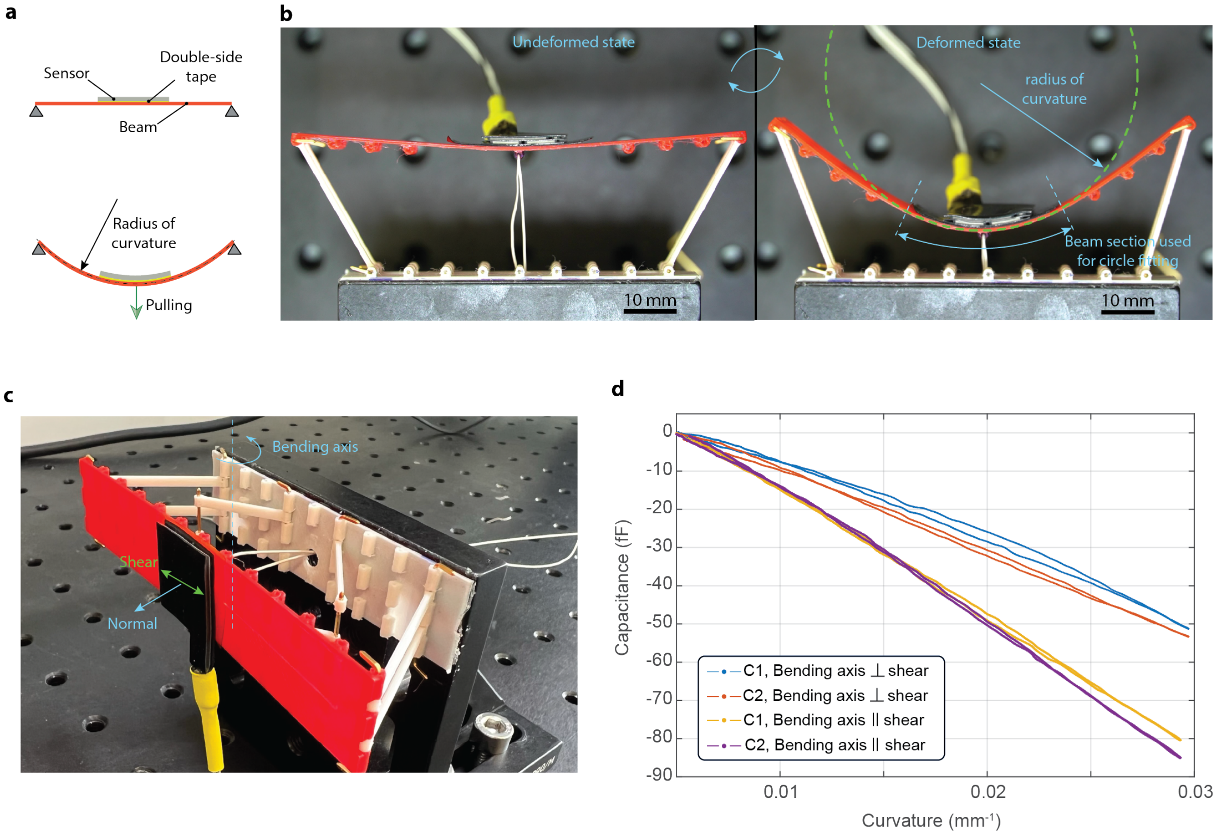

Supplementary Figure 12**. Sensor sensitivity vs. bending deformation. a** Schematic of experimental setup for controlled bending of the sensor. **b** The photographs of the undeformed and bent configurations (curvature is 0.03 mm^{-1}) of the sensor. **c-d** The sensor is attached to a bendable beam in two different configurations: the bending axis is orthogonal to the shear direction (blue and red curves) and the bending axis is parallel to the shear direction (yellow and purple curves). The change of the capacitances as a function of the bending curvature for two different orientations.

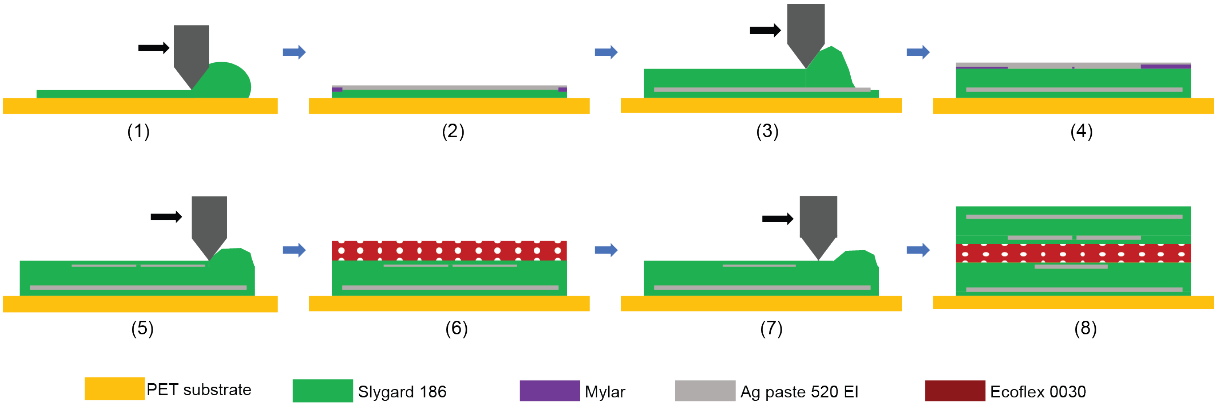

Supplementary Figure 8. **Fabrication process flow for silicone-foam sensors.**

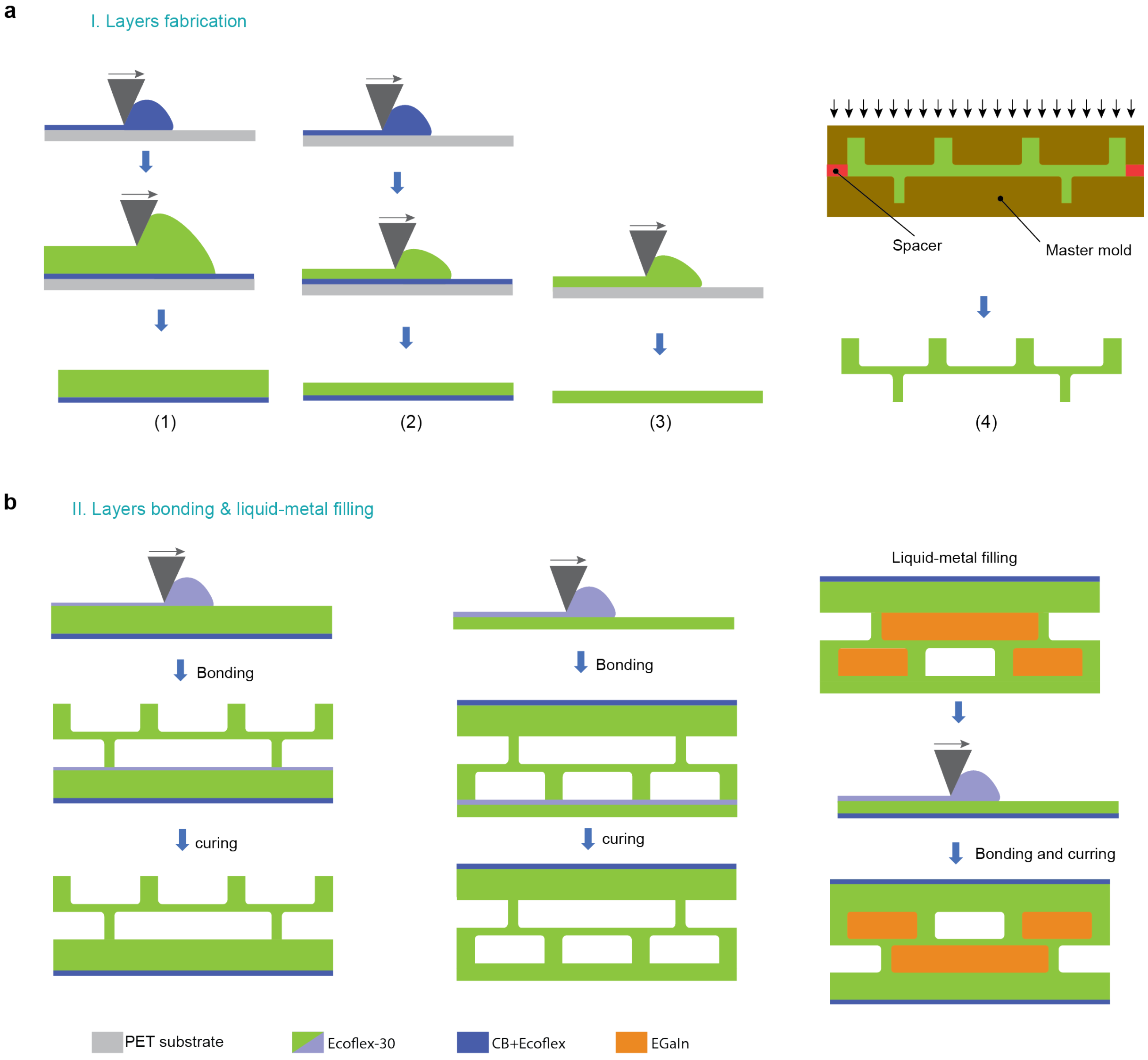

Supplementary Figure 9. **Fabrication process flow for liquid-metal sensors.**

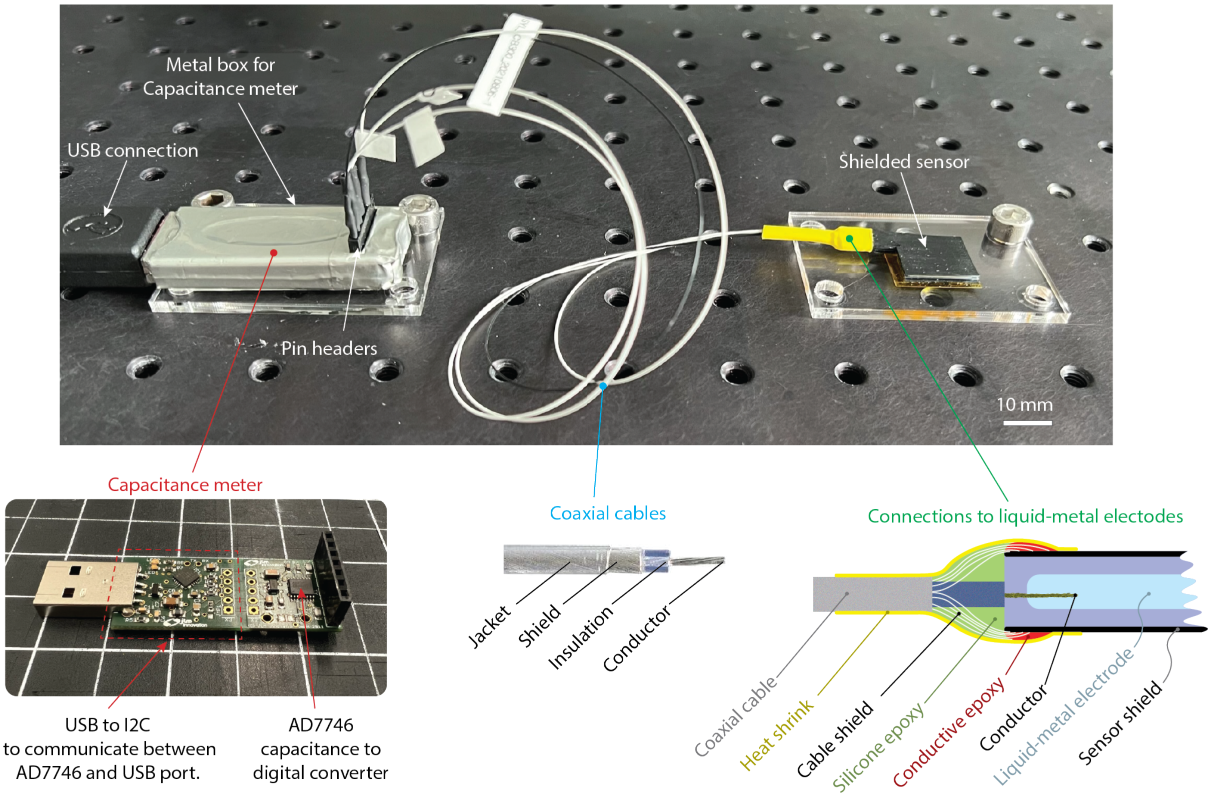

Supplementary Figure 15. **Electrical connections to the sensors using coaxial cables and the readout using a capacitance meter (Capmeter from JLM innovations GmbH)**. Here we show the connection of one coaxial cable to one sensing electrode. Each electrode is connected using a separate cable. The conductor of the coaxial cable is inserted into the LM channel and the cable shield (the ground terminal) is connected to the sensor shield. All connections are sealed with silicone epoxy and covered with heat shrink for mechanical robustness. The shielded cables carry the signal from the sensing electrodes to the AD7746 capacitance-to-digital (CDC) converter chip (from Analog Devices, Inc.) of the capacitance meter (from JLM innovation1). The CDC architecture used in the AD7746 measures the capacitance connected between the excitation pin and the input pin (floating capacitive sensing). In theory, any capacitance of less than 50 pF from these pins to ground should not affect the CDC output.

Supplementary References

[1] Baxter, L.K. Capacitive sensors: design and applications*. NY: IEEE Press* (1997).
