# Supplementary material for: Shielded soft force sensors
Source: Nat Commun. 2022 Aug 9;13:4649. doi: 10.1038/s41467-022-32391-0 (PMC9363457; doi:10.1038/s41467-022-32391-0)
Supplement: Supplementary file 3 — Description of Additional Supplementary Files [file 41467_2022_32391_MOESM3_ESM.pdf]

Description of Additional Supplementary Files

Supplementary Movie 1: Comparison between the sensing capacitances of the shielded and unshielded sensor in demanding testing scenarios: when a user's finger hovers over then sensors (not touching) and when generating sparks juts over the sensors.

Supplementary Movie 2: Demonstration of force measurement when handling soft objects. The soft sensor is mounted on a two-fingered robotic gripper and is used to measure the normal and shear forces when a water balloon is grasped, lifted, rotated, and released.

Supplementary Movie 3: Demonstration of soft sensor as sensory skin to measure the interaction forces during various daily life tasks such as screwing bolts, peeling fruits, cutting with knife, typing, and cleaning.

Supplementary Movie 4: Using soft sensors to accurately measure the traction force between the paper strip and the electroadhesive patch.
